# BAGLS, a multihospital Benchmark for Automatic Glottis Segmentation

**DOI:** 10.1038/s41597-020-0526-3

**Published:** 2020-06-19

**Authors:** Pablo Gómez, Andreas M. Kist, Patrick Schlegel, David A. Berry, Dinesh K. Chhetri, Stephan Dürr, Matthias Echternach, Aaron M. Johnson, Stefan Kniesburges, Melda Kunduk, Youri Maryn, Anne Schützenberger, Monique Verguts, Michael Döllinger

**Affiliations:** 1 Division of Phoniatrics and Pediatric Audiology, Department of Otorhinolaryngology, Head and Neck Surgery, University Hospital Erlangen, Friedrich-Alexander University Erlangen-Nürnberg, Waldstraße 1, 91054 Erlangen, Germany; 2 Department of Head and Neck Surgery, David Geffen School of Medicine at the University of California, Los Angeles, Los Angeles, California USA; 3 Division of Phoniatrics and Pediatric Audiology, Department of Otorhinolaryngology, Munich University Hospital (LMU), Munich, Germany; 4 NYU Voice Center, Department of Otolaryngology – Head and Neck Surgery, New York University School of Medicine, New York, New York USA; 5 Department of Communication Sciences and Disorders, Louisiana State University, Baton Rouge, Louisiana USA; 6 European Institute for ORL-HNS, Department of Otorhinolaryngology and Head & Neck Surgery, Sint-Augustinus GZA, Wilrijk, Belgium; 7 Department of Speech, Language and Hearing sciences, University of Ghent, Ghent, Belgium; 8 Faculty of Education, Health and Social Work, University College Ghent, Ghent, Belgium; 9 Faculty of Psychology and Educational Sciences, School of Logopedics, Université Catholique de Louvain, Louvain-la-Neuve, Belgium; 10 Faculty of Medicine and Health Sciences, University of Antwerp, Antwerp, Belgium; 11 Department of Otorhinolaryngology and Voice Disorders, Diest General Hospital, Diest, Belgium

**Keywords:** Medical research, Anatomy

## Abstract

Laryngeal videoendoscopy is one of the main tools in clinical examinations for voice disorders and voice research. Using high-speed videoendoscopy, it is possible to fully capture the vocal fold oscillations, however, processing the recordings typically involves a time-consuming segmentation of the glottal area by trained experts. Even though automatic methods have been proposed and the task is particularly suited for deep learning methods, there are no public datasets and benchmarks available to compare methods and to allow training of generalizing deep learning models. In an international collaboration of researchers from seven institutions from the EU and USA, we have created BAGLS, a large, multihospital dataset of 59,250 high-speed videoendoscopy frames with individually annotated segmentation masks. The frames are based on 640 recordings of healthy and disordered subjects that were recorded with varying technical equipment by numerous clinicians. The BAGLS dataset will allow an objective comparison of glottis segmentation methods and will enable interested researchers to train their own models and compare their methods.

## Abstract (summary)

**Table:** 

Measurement(s)	glottis • Image Segmentation
Technology Type(s)	Endoscopic Procedure • neural network model
Factor Type(s)	age • sex • healthy versus disordered subjects • recording conditions
Sample Characteristic - Organism	Homo sapiens

Machine-accessible metadata file describing the reported data: 10.6084/m9.figshare.12387890

## Background & Summary

Disorders of the human voice have a devastating impact on the affected and society in general. Numerous studies have shown a reduced quality of life^1^, a severe negative socioeconomic impact^2^ and the high prevalence of such disorders^3^. In particular, voice disorders have been associated with a variety of factors. For example, they are more prevalent in the elderly, where muscle atrophies and other age-related changes become a problem^4^. They also, on average, are more prevalent in women and some professions such as teachers and singers^3,5,6^.

**Fig. 1:**
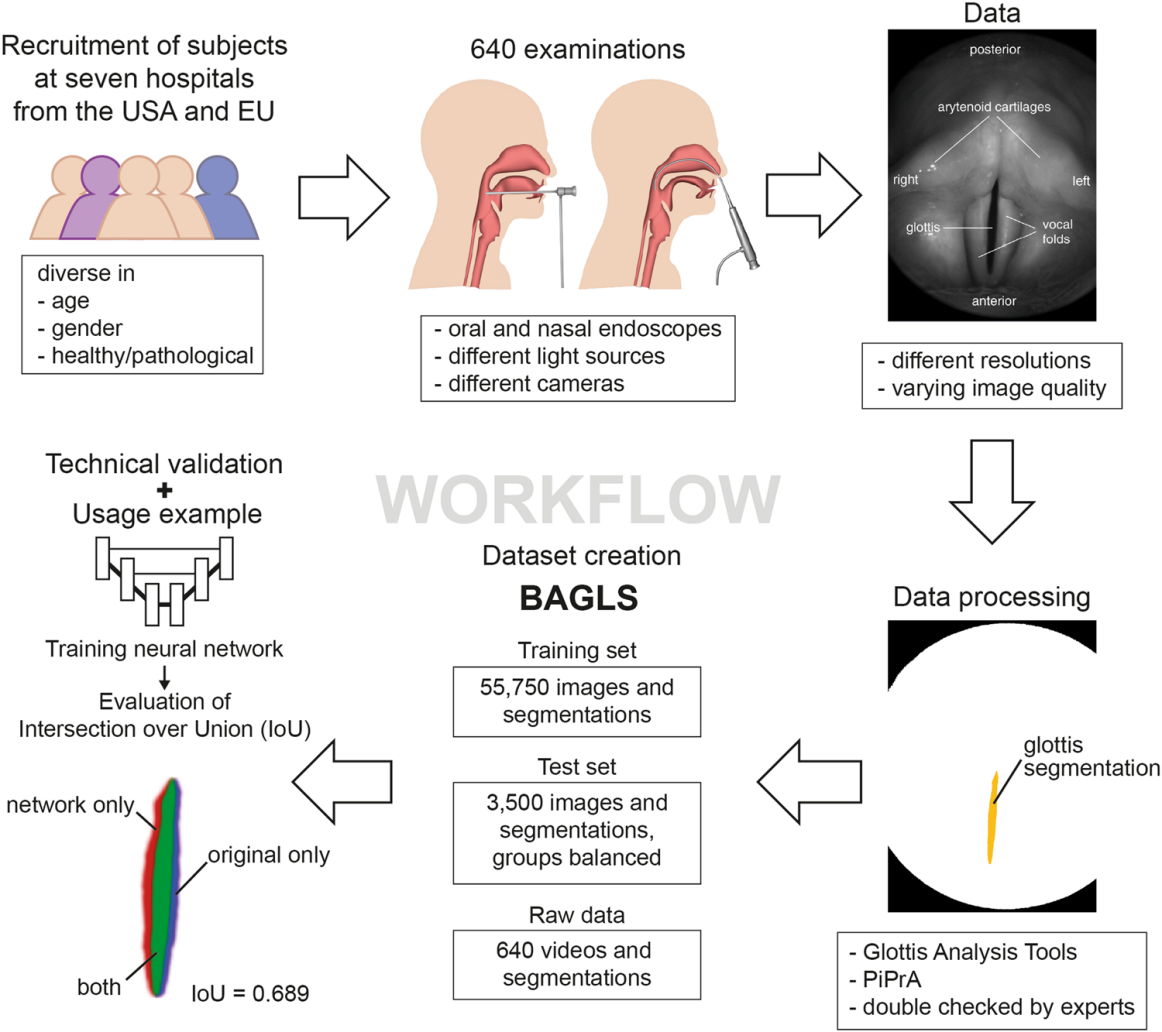
Workflow for creating the BAGLS dataset. Subjects with varying age, gender and health status were examined at different hospitals with differing equipment (camera, light source, endoscope type). The recorded image data is diverse in terms of resolutions and quality. Next, the glottis was segmented using manual or semi-automatic techniques and the segmentation was crosschecked. The segmented videos were split into a training and a test set. The test set features equal amounts of frames from each hospital. We validated BAGLS by training a deep neural network and found that it provides segmentations closely matching the manual expert segmentations.

Clinical examination of voice disorders is challenging due to the small-scale, high-frequency oscillation of the vocal folds which is critical in the creation of an acoustic speech signal^7^. Therefore, advanced imaging techniques, such as videostroboscopy and high-speed videoendoscopy (HSV)^8–10^, are employed clinically and in research. The involved anatomy and an exemplary endoscopic image are shown in Fig. 1.

**Fig. 2:**
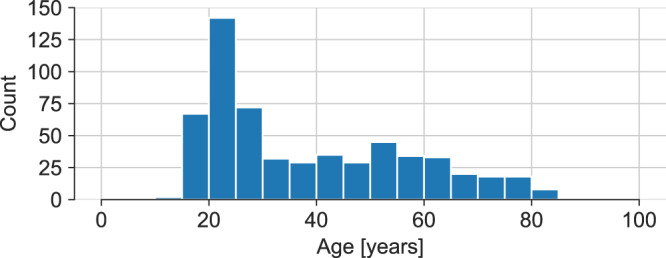
Age distribution of subjects in the BAGLS dataset.

One of the state-of-the-art methods in research and to enable a computer-aided diagnosis is the quantification of the vocal fold oscillation by segmenting the area between the vocal folds, the so-called glottis, in HSV recordings. A variety of oscillation parameters computed from the segmentation are used to provide an objective description of the oscillation^11–14^. However, the segmentation is still to some extent subjective, as it is usually performed semi-automatically with manual user intervention^15^. Even though automatic methods have been proposed^16,17^, they have only been evaluated on limited data from few individuals and, thus, offer limited comparability and transferability to other datasets. Overall, robust and automatic segmentations methods will reduce the workload of personnel and provide clinicians with more objective information than currently available in the clinical routine. Furthermore, such evidence-based diagnostics are critical in the health and insurance sector.

With the advent of deep learning methods, more robust yet fast image processing methods have become available^18^. However, they offer specific challenges as they require large amounts of annotated data^19,20^. Furthermore, these methods tend to require training on diverse datasets to allow transferability to new data^21^. Also, robustness is critical for a clinically-used deep neural network^22–25^. This is a challenge in medicine, where only limited data are available and the data usually require expert annotations. Additionally, data protection is particularly critical for medical data, which often limits the options for providing publicly available data. Furthermore, one has to ensure that the data cannot be deanonymized, i.e. allows for no derivation of any subject data. This is also the case in voice research and there are currently no public datasets for glottis segmentation. This is unfortunate, as in many domains, such as radiology^26–28^ or ophthalmology^29^, the availability of public datasets has propelled these topics to the frontier of machine learning research and spurred innovation, collaboration and research advances.

With the Benchmark for Automatic Glottis Segmentation (BAGLS), we aim to fill this gap and, in a collaboration of seven research groups from the USA and Europe, we created a benchmark dataset of HSV recordings for glottis segmentation. This multihospital dataset comprises recordings from a diverse set of patients, disorders and imaging modalities. It was annotated and double-checked by multiple experts. This dataset will allow an objective comparison of automatic segmentation methods, which will facilitate a more objective diagnosis, will save valuable expert time and is thus a crucial step in bringing automatic glottis segmentation to the daily clinical routine. We provide the BAGLS dataset to other research groups openly online and hope that it will fuel further advances and support international collaboration in the voice, medical imaging and machine learning community. Overall, this dataset fills the gap caused by the overall lack of a publicly available dataset for glottis segmentation and can serve as a litmus test for future methods for the task.

## Methods

The BAGLS dataset aims to provide a baseline as robust as possible. Therefore, it was created in such a way that it contains diverse samples from a variety of data sources, which are explored in detail in this section. Further, several experts created the segmentation masks for the data using specifically developed software tools for the task. To provide a baseline score and validate the benchmark data, we trained a state-of-the-art deep learning segmentation network on BAGLS. An overview of the acquisition, processing and validation steps is provided in Fig. 1.

### Videoendoscopy and Glottis Segmentation

Several imaging techniques have been conceived for recording the high-frequency, small-scale oscillation of the vocal folds and laryngeal endoscopy is one of the primary diagnostic tools for voice disorders^30^. The most common techniques are videostroboscopy^8^, videokymography^31^ and high-speed videoendoscopy (HSV)^10^. Videos are then inspected by clinicians to gain insight to aid diagnosis or, in research, to understand the phonatory process.

Segmentation of the glottal area has been a well-established practice to quantify the vocal fold oscillation and extract additional information from HSV recordings^32^.

Numerous studies have shown significant relationships between different disorders and parameters computed from the segmentation data^33–35^, such as the cepstral peak prominence^11^. Typical signals derived from the glottis segmentation are the glottal area waveform (GAW)^36^, the vocal fold trajectories^37^ and the phonovibrogram^38^. Parameters computed from these signals bear the promise of a higher objectivity than many of the purely subjective metrics still being employed in the clinical routine^36,39–41^. Figure 1 shows an exemplary HSV frame and the corresponding segmentation.

Even though the utility of glottis segmentation is clear, it is a laborious and time-consuming task that requires trained experts. And, although the binary segmentation into the classes background and glottal area might seem rather simple, in practice, there are several factors impeding completion of the task:

- Videos often feature a reduced image quality due to the technical requirements of HSV, such as a lower resolution and brief exposure time due to the high sampling rate^9^.
- Videos are often ill-lit, affected by patient movement and artifacts such as reflections caused by mucus and thus require additional image processing^32,42^.
- Parts of the glottis are often concealed due to the spatial limitations and parts of the anatomy such as the arytenoid cartilages covering others.
- Video quality and features vary noticeably depending on recording setup and subject.

Trained experts anecdotally require about 15 minutes to segment a 1,000 frames long HSV recording using specifically developed software. Therefore, several previous works have explored the possibility of performing an automated segmentation of the glottal area^16,17,43–46^.

However, all of the previous works only tested their methods on small datasets consisting of less than 25 different recordings from a single source, most used 10 or fewer HSV recordings. Further, common semantic segmentation metrics, such as the Dice coefficient^47^ or Jaccard index^48^ (also known as Intersection-over-Union), were often not determined.

The BAGLS benchmark dataset will be essential in testing segmentation algorithms’ practical applicability as it:

- provides the data necessary to train state-of-the-art deep learning methods for the task,
- allows an objective quantification of the quality of automatic segmentation methods,
- provides the data diversity necessary to achieve robustness in the clinical routine, where algorithms that are trained on data from one source usually do not perform well on data from another source.

### Data

The performance of deep learning methods usually only translates to data from similar sources. Transfer between different data sources can be achieved using transfer learning techniques, but is not guaranteed^21,49^. Videoendoscopy is a widely employed imaging modality, and, thus, the differing recording hardware, software and varying clinicians introduce great variability to the data. To ensure that an automatic segmentation method actually performs well on the whole range of data, it is essential to also test it on a diverse dataset. This is one of the core motivations of this work and the provided data show a great diversity in the following respects:

- The HSV recordings were collected at seven different institutions from the USA and Europe.
- The data were collected using a variety of cameras, light sources, image resolutions, sampling rates and endoscope types.
- The data contain samples with both healthy and disordered phonation, presenting with both functional and organic dysphonia.
- The dataset is comprised of recordings from all age groups except young children and contains large amounts of samples from male and female subjects.
- The data contain pre-dominantly grayscale, but also color images (RGB).

Ethnicity was not determined during data collection. Thus, we presume the data reflects the average ethnicity distribution at the respective hospitals. Notably, the ethnic background may have an influence on the parameters derived from the segmentation^50,51^.

In this study, we not only provide the first publicly available dataset for glottis segmentation but also one that reflects the diversity of the clinical reality and is therefore particularly well suited to measure the performance of segmentation methods for this task.

All data were acquired in accordance with local ethics committees and are covered by Boston University IRB (2625), University of Illinois, Urbana-Champaign, IRB (14141), Ethikkommission University Hospital Freiburg (EK83/15), University of Ghent (190311RETRO), Louisiana State University IRB (2668), Medical School at Friedrich-Alexander-University Erlangen-Nürnberg (290_13B), and the University of California, Los Angeles, (2010-021-(01, 02, 02A, 03, 03A, 11, 12, 13, 21, 21A, 22, 23, 23A, 31)). Written consent was obtained from all subjects.

#### Data Diversity

In deep learning, which is used for state-of-the-art segmentation algorithms^19,20^, data diversity is also critical as the trained networks usually reflect potential biases in the data, such as are also known to be problematic as the trained networks usually reflect potential biases in the data^52^. In case of glottis segmentation, this is comparatively less problematic as the methods are not aimed to provide a supposed diagnosis and possible errors in the segmentation can be spotted relatively easily. However, to ensure that the trained networks perform well on the broad range of recorded data and for underrepresented cases it is necessary to include at least some of these cases in the data. Note that the dataset thus explicitly aims to cover diverse image acquisition modalities and, by design, avoids standardization of the image acquisition procedure which inherently varies between hospitals and countries. We aimed to achieve the necessary diversity by working together in an international cooperation, where each group specifically aimed to provide data matching the diversity encountered in their clinical and research routine. An overview of the number of videos and frames per group in the training and test is given in Table 1. As the availability of data differs between groups, it was not possible to balance the training data such that each group is represented equally. The test data, however, is split equally among groups ensuring that a method has to perform well on data from all or most of the institutions to achieve good scores on the benchmark. The noticeable overrepresentation of training data from the Erlangen group may thus influence training, but the scores on the balanced test set will indicate if this was a problem.

**Table 1:** Composition of the dataset in relation to origin; the training data featured 50 or 100 frames per video depending on video length and test data 50 frames per video.

Institution	# in training	# in test
Boston University	10	10
Louisiana State University	15	10
New York University	14	10
Sint-Augustinus Hospital, Wilrijk	30	10
University of California, Los Angeles	20	10
University Hospital Erlangen	458	10
University Hospital of Munich (LMU)	23	10
Total Number of Videos	570	70
Total Number of Frames	55750	3500

**Table 2:** Overview of voice disorders represented in the BAGLS dataset, multiple disorders per video are possible.

Disorder Status	# of videos	Disorder Status	# of videos
Healthy	380	Contact granuloma	5
Muscle tension dysphonia	139	Paresis	4
Muscle thyroarythaenoideus atrophy	25	Laryngitis	4
Vocal insufficiency	18	Papilloma	1
Edema	14	Leucoplacia	1
Insufficient glottis closure	14	Carcinoma	1
Nodules	13	Other	8
Polyp	9	Unknown status	50
Cyst	6

**Table 3:** Overview of the sampling rates and resolutions of the recorded HSV data in the dataset.

Sampling rate [Hz]	# of videos	Resolution	# of videos
1000	21	256 × 120	15
2000	17	256 × 256	88
3000	30	288 × 128	7
4000	542	320 × 256	33
5000	1	352 × 208	30
6000	2	352 × 256	11
8000	26	512 × 96	1
10000	1	512 × 128	22
		512 × 256	431
		512 × 512	2

We provide individual frames of the videos that are discontinuous and randomly selected to enhance data diversity as consecutive frames typically show little variation. For each video in the test dataset, 50 frames were randomly selected leading to a total of 3500 frames from 70 videos. For the training data, either 50 or 100 frames (some videos were too short for more than 50 discontinuous frames) were randomly selected and a total of 55750 frames was selected from 570 HSV recordings. We further provide the entire raw data used to create the BAGLS dataset. With this, algorithms based on time variant data can be trained and analyzed as suggested by^17^ or used in studies focusing on kymography^53,54^.

We provide a detailed breakdown of the provided data in terms of age, sex and disorder status to emphasize the data diversity and give a detailed overview of the data. The frames and videos contained in the BAGLS dataset are provided with corresponding metadata. Figure 2 shows the age distribution in the data. The mean age was 38.22 ± 18.58 years, with a range from 14 to 91. The data stem from 432 females and 177 males, for 31 recordings no sex was reported. In Table 2 diagnosed voice disorders at the time of the recording are shown. In total, 380 recordings of healthy subjects, 262 cases of identified disorders and/or noticeably affected vocal fold oscillations are present in the data. No health status was available for 50 subjects. Notably, disorders are heterogeneous and besides functional disorders also include organic disorders, such as edema as well as benign and malignant neoplasias. Recordings including abnormal tissue growth may lead to poor segmentations and incorporation of those recordings in the dataset is crucial for judging the automatic segmentation methods’ robustness.

Overall, the dataset features great variability and diverse representation in terms of age, sex, and disorder status. Furthermore, as it comprises data from seven institutions, a multitude of clinicians were involved in the acquisition of the recordings, which further broadened the diversity of the dataset.

#### Technical Equipment

**Fig. 3:**
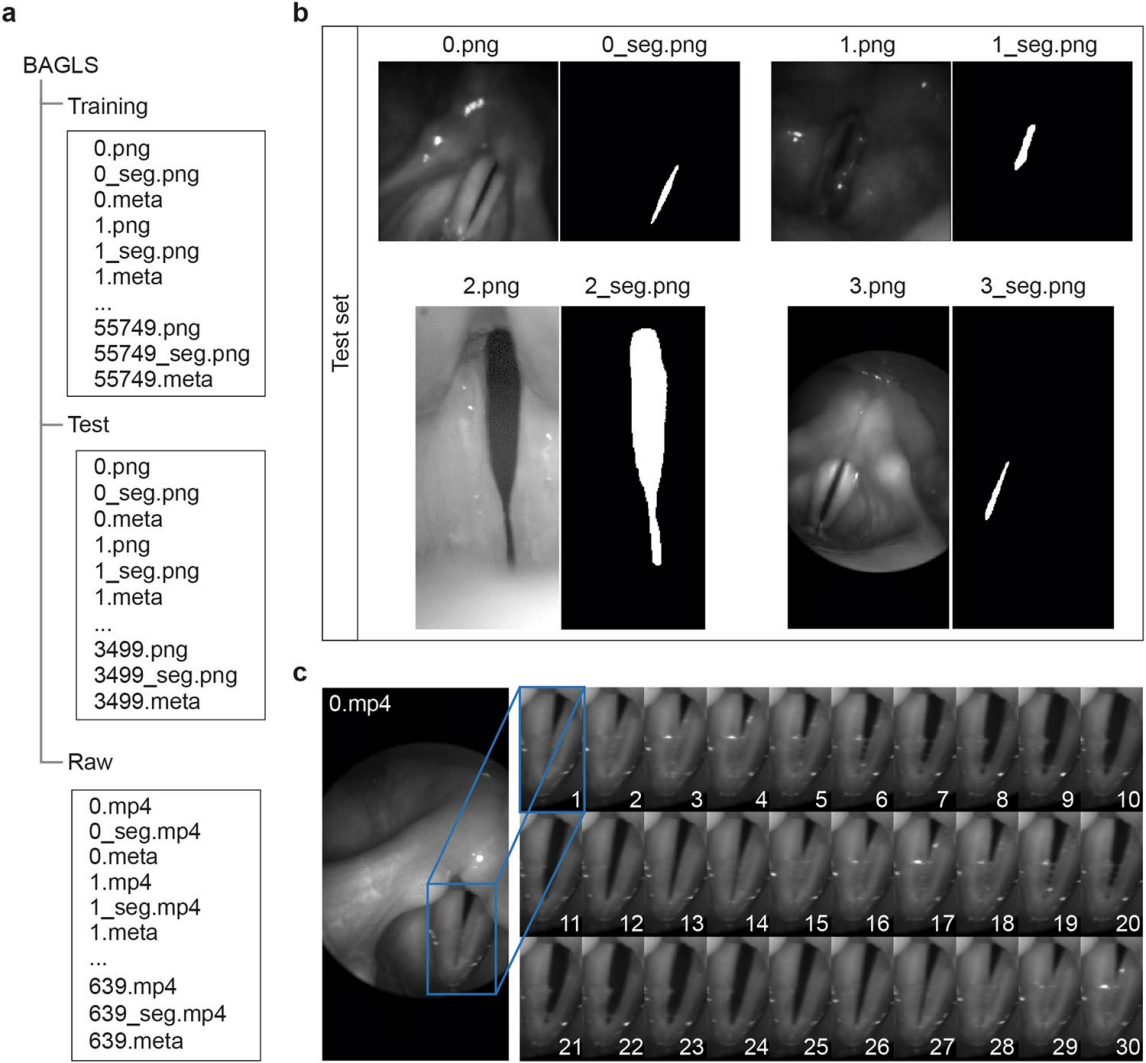
The BAGLS dataset. (**a**) The folder structure of the dataset. We provide two folders for training and test, respectively, containing image/segmentation pairs, and one folder that contains the raw data (folder “raw”). (**b**) Exemplary images from the dataset next to the binary segmentation mask as present in the folders shown on the left. Note differing aspect ratios and image properties such as black image borders. (**c**) An exemplary subset from raw video 0.mp4 where two open/close cycles are visible. For illustration purposes, we cropped the image (blue frame). Consecutive frame ids (1…30) are in the lower right corner.

The dataset does not only feature diversity in regard to clinicians and subjects involved. As the different institutions represented in this work use differing equipment and recording setups, a great variety of cameras, light sources, endoscopes and imaging settings, such as sampling rate and image resolution, are represented. Utilized sampling rates range from 1000 Hz to 10000 Hz and are shown in detail in Table 3 on the left side. The different image resolutions in the dataset can be seen on the right side of Table 3. In particular, the smallest resolution is 256 × 120 px and the largest one 512 × 512 px. Note that aspect ratios of the images also vary (see Fig. 3). Tables 4 and 5 provide an overview of the utilized cameras, light sources and endoscope types. Five different cameras were used for the HSV recordings with three different light sources. The dataset contains recordings acquired with rigid oral endoscopes at an angle of 70° and 90° as well as flexible nasal endoscopes with two different diameters. Overall, 618 videos contained grayscale data and 22 featured RGB data.

**Table 4:** Overview of cameras used to record the HSV.

Camera	# of videos
KayPentax HSV 9700 (Photron)	16
KayPentax HSV 9710 (Photron)	495
HERS 5562 Endocam Wolf	79
Phantom v210	30
FASTCAM Mini AX100 540K-C-16GB	20

**Table 5:** Overview of the utilized light sources and endoscopes to record the HSV data.

Endoscope type	# of videos	Light source	# of videos
Oral 70°	543	Kay Pentax Model 7152B	491
Oral 90°	46	Xenon Light	
Nasal 2.4 mm	9	Wolf 300 W Xenon	79
Nasal 3.5 mm	12	CUDA Surgical E300 Xenon	40
N/A	30	N/A	30

**Table 6:** Example metadata for a given frame (BAGLS test/0.meta).

Key	Value
Video Id	27
Camera	HERS 5562 Endocam Wolf
Sampling rate (Hz)	4000
Video resolution (px, HxW)	[256, 256]
Color	false
Endoscope orientation	70°
Endoscope application	oral
Age range (yrs)	90–100
Subject sex	f
Subject disorder status	spasmodic dysphonia
Segmenter	1
Post-processed	2

**Table 7:** Technical Validation of External Expert Segmentations.

Additional Expert Id	IoU
E1	0.745
E2	0.749
E3	0.798
E4	0.796
Average	0.772

### Expert Annotations

Three experts in glottis segmentation created the segmentations for the dataset. Previous studies have shown that inter- and intra-rater variability in voice research can be a concern^55–58^. The BAGLS dataset aims to compensate for this by using segmentations that were crosschecked by multiple experts. We further validated these segmentations as described in the Technical Validation section. Two different software tools were used to ensure a high quality of the segmentation mask, especially in the test data. The detailed segmentation procedure was as follows:

1. Videos were inspected to judge which software tool, either the *Glottis Analysis Tools* (GAT) software or the *Pixel-Precise Annotator* (PiPrA) software, was appropriate for the segmentation (both are described in the following).
2. Each video was segmented by one expert using the selected software.
3. After an additional inspection of the video, the segmentation was either refined using the PiPrA software or kept as is.
4. After segmentation of all videos, videos were randomly split into test and training sets so that each group contributed ten videos to the test data and the rest to the training data.
5. As scores rely on the test data segmentations, they were checked once by another expert and, when necessary, adjustments were made using the PiPrA software.

#### Glottis Analysis Tools

One of the two software tools used to create the annotated samples, i.e. the segmentation masks for the data, is the GAT software developed in-house by the Erlangen group. The software is well known in the field and utilized by several renowned research groups to analyze HSV data^13,58–60^. The software provides a semi-automatic method to create segmentation masks using a seed-based region growing algorithm that relies on grayscale thresholds. In some cases, video quality was however insufficient - especially in regards to lighting - to allow a segmentation using GAT. In those cases the PiPrA software was employed. GAT is typically also used to compute the mentioned voice parameters such as the CPP^36^, but in our case we only required the segmentation masks. The GAT software is available on request (http://www.hno-klinik.uk-erlangen.de/phoniatrie/forschung/computational-medicine/gat-software/) and can be obtained from the research group in Erlangen.

#### Pixel-Precise Annotator

As the GAT software is streamlined for a rapid semi-automatic processing of HSV data, it currently does not support individual pixel adjustments. Furthermore, it is targeted at processing videos and not individual, discontinuous frames. Therefore, we developed a new software targeted specifically at creating high-quality, pixel-precise segmentation masks for the generation and fine tuning of the BAGLS data. The software implements a flood fill algorithm, but also allows annotating individual pixels. It can perform basic preprocessing steps such as brightness and contrast adjustments as well as a CLAHE histogram equalization. This software was utilized to annotate cases where the videos were of particularly low quality or featured insufficient illumination for the segmentation with GAT. The PiPrA software was also used for the final two checks by the experts to ensure that the test data met highest standards so that test scores are as reliable as possible. We provide the software open source online (https://github.com/anki-xyz/pipra).

## Data Records

We made BAGLS online available at Zenodo (^61^, 10.5281/zenodo.3377544, link to latest version) and at Kaggle (https://www.kaggle.com/gomezp/benchmark-for-automatic-glottis-segmentation). Also, we provide an interface to the individual data to preview and select a subsection of the data at https://www.bagls.org.

As described in the Methods section, it consists of a blend of data from seven institutions, split into training and test set, and the raw data (Fig. 3a). In Fig. 3b example pairs of videoendoscopic images and corresponding binary segmentation masks from the test set are shown. As PNG files the data can be viewed using standard image software, and also imported for image processing into other software, such as Python and MATLAB, to develop and evaluate new segmentation methods.

We further provide the raw data as videos in *.mp4 file format with very high quality encoding settings (example see Fig. 3c). The raw data can be previewed online at www.bagls.org as video snippets with 30 consecutive frames. Mp4 files can be opened with any conventional video playback software, such as VLC Media Player or QuickTime. For each raw video, we provide the corresponding segmentation maps created by our baseline neural network (see later paragraphs).

Every file is accompanied by a JSON (JavaScript Object Notation) file that contains important metadata related to the recording. This file is human-readable and can be opened with a standard text editor, as well as dynamically opened and processed in common programming languages, such as MATLAB or Python. In Table 6, we show the metadata for an example file re-arranged for illustration purposes.

## Technical Validation

We inspected all of the available videos to ensure they contains no fragmented or any corrupted frames. However, we intentionally kept frames that are ill-lit, don’t show the vocal folds and/or the glottis, or are contaminated with recording artifacts, such as a honey comb pattern in case of recordings with flexible endoscopes. The reason for this is that any method will have to deal with these problems in a real clinical setting as well. The entire BAGLS dataset itself was also inspected twice by different segmenters. In the metadata that is shipped with the BAGLS dataset, we state the ID of the segmenter that originally segmented the frame (in the field “segmenter”, 0,1 or 2 respectively) as well as the segmenter that checked and optionally post-processed the frame (“post-processed”, same id assignment as “segmenter”).

The manually segmented frames comprise a key component of the BAGLS dataset. As the frames are segmented by individuals and the image quality varies across recordings and especially pixels at borders are hard to classify the ground-truth is somewhat subjective (see earlier paragraphs). To validate the segmentation quality, we obtained another 500 segmentations by four other segmentation experts based on the same 500 randomly chosen frames from the BAGLS dataset and compared the *Intersection over Union* (IoU)^48^ metric that describes the overlap of two segmentations, e.g. from expert E1 and the ground-truth of a given frame:

$${\rm{IoU}}(A,B)=\frac{A\cap B}{A\cup B},$$

with *A* and *B* being two binary images classifying each pixel into foreground (1, glottis) and background (0, not glottis). The IoU score ranges between 0 (no overlap at all between *A* and *B*) and 1 (perfect overlap between *A* and *B*). We provide the median IoU scores for each additional expert in Table 7.

This resulted in an average IoU score of 0.772 indicating a high overlap across experts. As recently shown, glottis segmentation is inherently subjective^58^, although we believe that the three experts providing the ground-truth ensured being as objective as possible.

## Usage Notes

The dataset is accessible in several ways. On Zenodo and Kaggle, it is provided as three zip files that contain the training, the test and the raw data, respectively. The training and test folder contain 55,750 and 3,500 pairs of high-speed videoendoscopy frames and their respective binary segmentation masks with the corresponding metadata, respectively. The files are saved as losslessly compressed PNG files and can thus be opened using conventional image readers. Benchmark scores should be reported on the data from the test folder, which should not be included during training. The metadata is in JSON file format and can be opened using conventional text editors. The raw data folder (“raw”) contains 640 files in mp4 file format and can also be opened using conventional video playback software. Additionally, each raw data entry is accompanied by corresponding segmentation maps created by our baseline neural network (see later paragraphs).

To demonstrate the applicability of the dataset - the ultimate goal of the BAGLS dataset -, we trained a deep neural network to perform the segmentation task. We used the *U-Net* architecture, a fully convolutional neural network architecture introduced by Ronneberger *et al*.^62^. It became popular, especially in medical imaging, after winning the ISBI cell tracking challenge 2015. It is characterized by its similarity to autoencoder architectures as it features an encoding and decoding part in the network. However, it also utilizes skip connections between blocks of the same (or close) spatial dimensions in the encoder and decoder part. It has seen application to a variety of problems in medical imaging^19^.

### Preprocessing

We applied several preprocessing steps to the BAGLS dataset for training the U-Net. We supply the unmodified data so that other researchers may test different preprocessing steps suitable to the architectures and approaches they might choose.

First, as most of the data are grayscale and color information is not inherently relevant to the segmentation task, we converted all images to grayscale. Secondly, as training batches in the Keras deep learning framework that we used, may not contain different image resolutions, we resized the training data to a resolution of 512 × 256 pixels. For the test data, images that had resolutions that were not multiples of 32 were zero padded until the resolution was divisible by 32, as this was necessary for the used architecture due to the intra-network pooling operations. Pixel intensities (*I*) were normalized to ${I}_{normalized}\in [-1,1]$ using ${I}_{normalized}=\frac{I}{127.5}-1$.

To improve generalization capabilities of the trained models, several data augmentation methods were used. In particular, we used the *albumentations* Python package^63^ to apply random brightness, contrast, gamma, Gaussian noise and blurring changes and also random rotations and horizontal flips to the images. This was done asynchronously during training. Thus, each training epoch featured novel training data.

### Setup

Training and inference were performed on a Titan RTX graphics card using TensorFlow 1.13.1^64^ with Keras 2.2.4. We employed the Adam optimization algorithm^65^ with a cyclic learning rate between 10^−3^ and 10^−6^ ^66^. The model was trained on a dice loss function. Training ran for 25 epochs.

To assess neural network performance, we investigated the common IoU metric as introduced in the Technical Validation section. However, instead of comparing segmentations of different experts, we here compare the predicted segmentation to the respective ground-truth as provided in the BAGLS dataset.

The training data were split into a training and validation set of 52,962 and 2,788 images respectively to track possible overfitting without evaluating the test data. The model with the highest IoU score on the validation set was chosen for subsequent analyses. The test set was evaluated only once for the final model.

### Validation

**Fig. 4:**
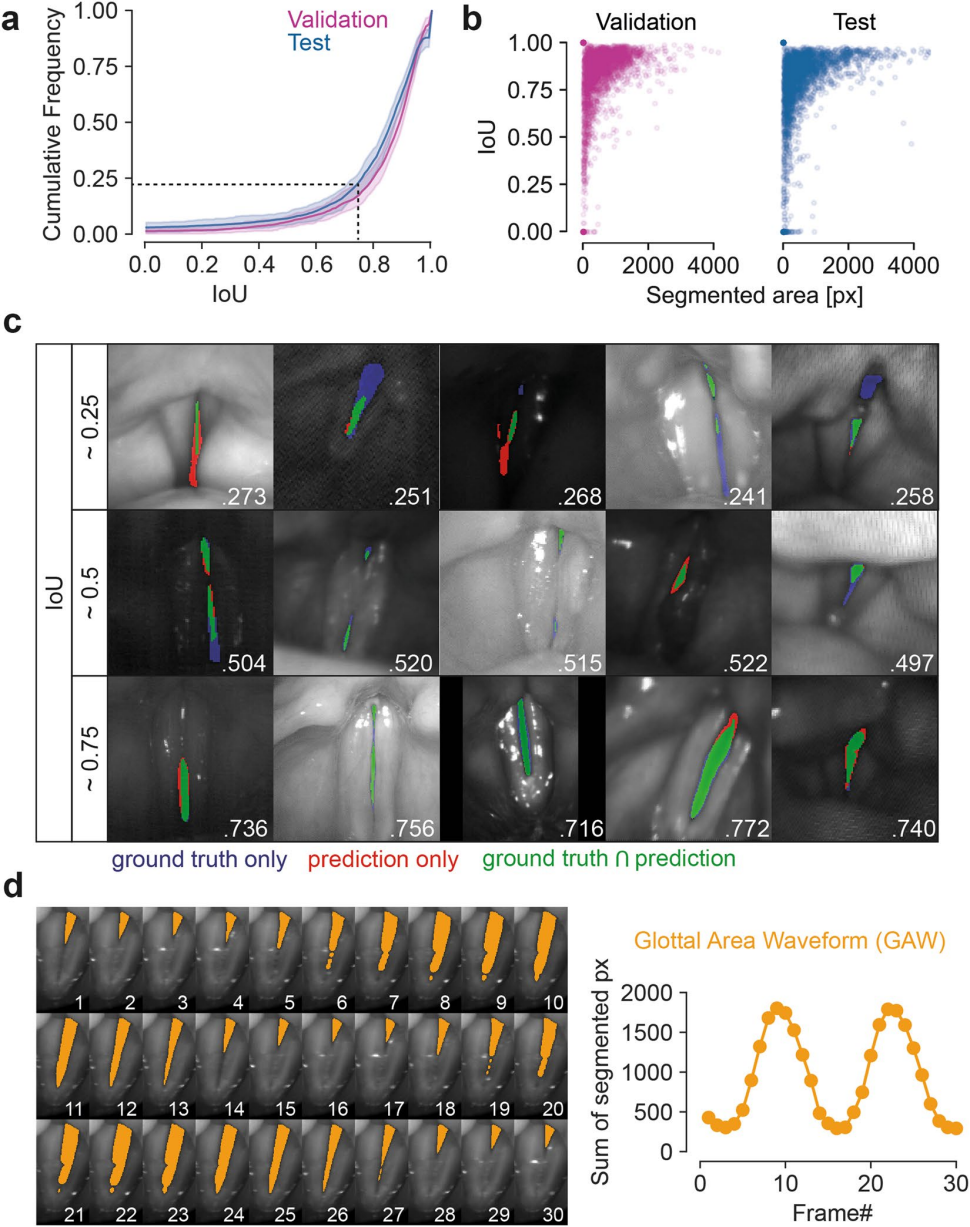
Evaluation of model performance using the Intersection over the Union (IoU). (**a**) Cumulative distribution of the IoU across validation (magenta) and test (blue) set. Shaded error shows 95% confidence interval of bootstrapped distributions. (**b**) Distribution of IoUs against segmented area in the ground truth. Left: validation set; right: test set. (**c**) Example images and segmentations for IoUs close to 0.25, 0.5 and 0.75. Intersection of segmented pixels in the ground truth and prediction in green. Blue and red pixels were classified as glottis only in the ground truth and only in the prediction, respectively. (**d**) An example video from the BAGLS dataset (0.mp4, same subset as in Fig. 3c) segmented (orange overlay) using the trained model with respective glottal area waveform (sum of segmented pixels over time).

After 21 training epochs, a maximum IoU of 0.831 was reached on the validation set. The model achieved an IoU score of 0.799 on the test set. Figure 4a shows cumulative IoU scores on the test and validation set. The distribution is skewed and, thus, the mean is proportionally more affected by the lower IoU scores. Notably, the overrepresentation of data from the Erlangen group in the training data does not seem to be particularly detrimental. This is clearly visible in Fig. 4a as the distribution of IoU scores for both, validation and the balanced test set, are very similar. As the sample sizes are quite large and already tiny differences would be statistically significant, we decided to use bootstrapping, a common way to resample a given distribution and provide a confidence interval^67^. As the 95% confidence intervals are highly overlapping, we assume that both, validation and test test are drawn from the same distribution.

Figure 4b shows the distribution of IoU scores against the segmented area in the ground truth. Especially smaller areas correlate clearly with lower IoU scores. This is expected as the decision if individual pixels belong to the glottis can be particularly difficult and is score-wise proportionally more impactful for smaller areas. For example, if the ground-truth area consists only of three pixels, and the prediction consists of these very same three pixels, but also contains one more pixel, the IoU is 0.75. If the ground-truth area consists, however, of 100 pixels, and the prediction contains one more pixel not included in the ground-truth, the IoU is 0.99.

Over 75.8% of the test set segmentations have an IoU greater than 0.75, which already resembles a high segmentation quality (dashed line in Fig. 4c). We also assessed the runtime of our model. Each forward pass took 24 ms on on a graphics processing unit (GPU) or 2.12 s on the central processing unit (CPU). Thus, the analysis of a 500 frame video (resolution 512 × 256 px) requires about 12.0 s and 17.7 min for GPU and CPU, respectively.

We further evaluated the performance of the trained model on a coherent Video In Fig. 4d we show the segmentation performance on each single frame and the resulting glottal area waveform. We used this baseline neural network to segment each frame of the entire raw data and provide these segmentations together with the corresponding raw data online.

We provide a complete example of utilizing BAGLS online (https://github.com/anki-xyz/bagls). It features loading and preprocessing the data, training a deep neural network and segmentation of an example video.

## Data Availability

We provide the Glottis Analysis Tools software on request (http://www.hno-klinik.uk-erlangen.de/phoniatrie/forschung/computational-medicine/gat-software/). The Pixel-Precise Annotator tool (PiPrA) is available open source online (https://github.com/anki-xyz/pipra). We provide a Jupyter notebook for training, evaluating and using the deep neural network as used in the Usage Notes section online under an open source license (https://github.com/anki-xyz/bagls).
